# Phytotoxic Activity and Identification of Phytotoxic Substances from *Schumannianthus dichotomus*

**DOI:** 10.3390/plants9010102

**Published:** 2020-01-14

**Authors:** Md. Mahfuzur Rob, Kawsar Hossen, Arihiro Iwasaki, Kiyotake Suenaga, Hisashi Kato-Noguchi

**Affiliations:** 1 Department of Applied Biological Science, Faculty of Agriculture, Kagawa University, Miki, Kagawa 761-0795, Japan; hisashi@ag.kagawa-u.ac.jp; 2 The United Graduate School of Agricultural Sciences, Ehime University, 3-5-7 Tarumi, Matsuyama, Ehime 790-8566, Japan; 3 Department of Chemistry, Faculty of Science and Technology, Keio University, 3-14-1 Hiyoshi, Kohoku, Yokohama 223-8522, Japan; a.iwasaki@chem.keio.ac.jp (A.I.); suenaga@chem.keio.ac.jp (K.S.)

**Keywords:** *Schumannianthus dichotomus*, phytotoxic substances, growth inhibition, syringic acid, methyl syringate

## Abstract

The phytotoxic potential of plants and their constituents against other plants is being increasingly investigated as a possible alternative to synthetic herbicides to control weeds in crop fields. In this study, we explored the phytotoxicity and phytotoxic substances of *Schumannianthus dichotomus*, a perennial wetland shrub native to Bangladesh, India, and Myanmar. Leaf extracts of *S. dichotomus* exerted strong phytotoxicity against two dicot species, alfalfa and cress, and two monocot species, barnyard grass and Italian ryegrass. A bioassay-driven purification process yielded two phenolic derivatives, syringic acid and methyl syringate. Both constituents significantly inhibited the growth of cress and Italian ryegrass in a concentration-dependent manner. The concentrations required for 50% growth inhibition (*I*_50_ value) of the shoot and root growth of cress were 75.8 and 61.3 μM, respectively, for syringic acid, compared with 43.2 and 31.5 μM, respectively, for methyl syringate. Similarly, to suppress the shoot and root growth of Italian rye grass, a greater amount of syringic acid (*I*_50_ = 213.7 and 175.9 μM) was needed than methyl syringate (*I*_50_ = 140.4 to 130.8 μM). Methyl syringate showed higher phytotoxic potential than syringic acid, and cress showed higher sensitivity to both substances. This study is the first to report on the phytotoxic potential of *S. dichotomus* and to identify phytotoxic substances from this plant material.

## 1. Introduction

The success of agricultural production depends on the efficient management of different stress factors, including weed infestation. Around 7000 weed species have been recognized so far, and possibly a couple of hundred cause problems in agricultural production [1]. Using natural compounds to control weeds has long been accepted as an environmentally friendly approach, but during the last four decades, farmers have mostly relied on toxic synthetic agrichemicals [2]. However, in recent years, demand for organic farming has markedly increased all over the world. This system aims to grow crops using natural products rather than external inputs, enhancing the reduction of toxic residues and providing safe food [3,4]. To ensure sustainable organic farming, it is essential to use diversified techniques to control weeds rather than synthetic herbicides. Plant species capable of suppressing the growth of other plants are excellent resources for weed control and could revolutionize sustainable agriculture [5,6]. The ability of plants to suppress the growth of other plants is governed by their allelopathic potential [7,8,9]. Allelopathy is an interference mechanism in which plants or their dead parts release biochemicals known as allelochemicals that exert adverse effects (phytotoxicity) against the associated plants [10,11]. The investigation of phytotoxic potential provides useful clues in analyzing new models of natural herbicides [12,13]. Several plant materials and their identified allelochemicals have been reported to possess a satisfactory phytotoxic potency to act as natural herbicides [14,15]. In fact, some allelochemicals are already being used in biopesticide formulations [16,17].

*Schumannianthus dichotomus* (Roxb.) Gagnep, commonly known as ‘Murta’ in Bangladesh, belongs to the Marantaceae family [18,19,20]. The species is also found in India, Myanmar, Thailand, Vietnam, Malaysia, and the Philippines [21,22]. It is a shrub with dichotomously branched stems and grows up to 3–5 m tall with oblong, lanceolate leaves [23,24]. The shrub is a shade-loving plant and prefers soil with high moisture [25]. Nevertheless, it is commercially grown in the north-eastern parts of Bangladesh and in some parts of India [26]. A substantial proportion of the rural population in the Sylhet region of Bangladesh supports their livelihood by making handicrafts from *S. dichotomus* [27]. A long strip obtained from the bark of mature *S. dichotomus* is used as raw material for those handicrafts, especially for preparing traditional bed mats (known as ‘Sital pati’ in the local language) [28], which are durable and very comfortable during summer [29].

Some studies have revealed that there are no significant pests or disease infestations in fields growing *S. dichotomus* and that the plant can be cultivated with minimal or no intercultural operations including weeding [18,30]. The hardy nature of *S. dichotomus* attracted our attention, and we anticipated that this plant material might have some phytotoxic potential that allows successful growing conditions with minimal weeds growing around it. Although some research has been carried out on the phytotoxic potential of different species belonging to the Marantaceae family, to the best of our knowledge, there have been no reports on either the phytotoxic effect or the phytotoxic substances responsible for the phytotoxicity of *S. dichotomus*. Therefore, the aim of our study was to investigate the allelopathic potential of *S. dichotomus* leaves and the compounds responsible for its phytotoxicity.

## 2. Results

### 2.1. Phytotoxic Activity of S. dichotomus Leaf Extracts

**Figure 1:**
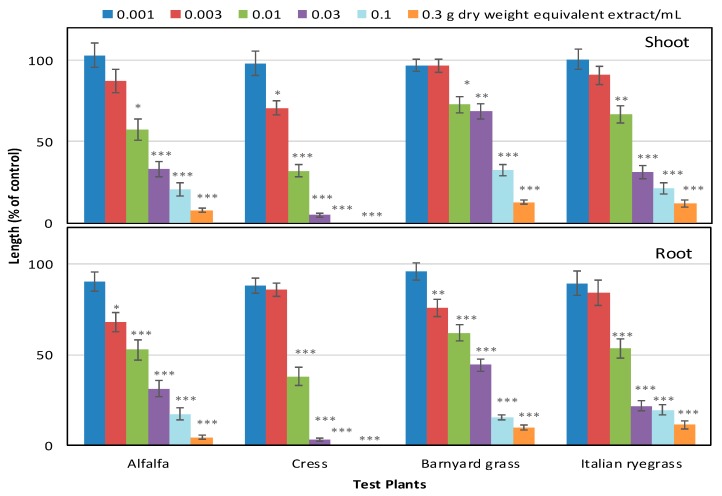
Effect of *Schumannianthus dichotomus* extracts on the shoot and root growth of alfalfa, cress, barnyard grass, and Italian rye grass. All the test plant species were treated at the concentrations of 0.001, 0.003, 0.01, 0.03, 0.1, and 0.3 g dry weight equivalent extract of *S. dichotomus*/mL. Values represent mean ± SE of two independent experiments with three replications for each treatment (number of seedlings per treatment = 10, n = 60). Each vertical bar represents standard error of the mean. Asterisks indicate significant differences between treatment and control: * *p* < 0.05, ** *p* < 0.01, and *** *p* < 0.001.

**Figure 2:**
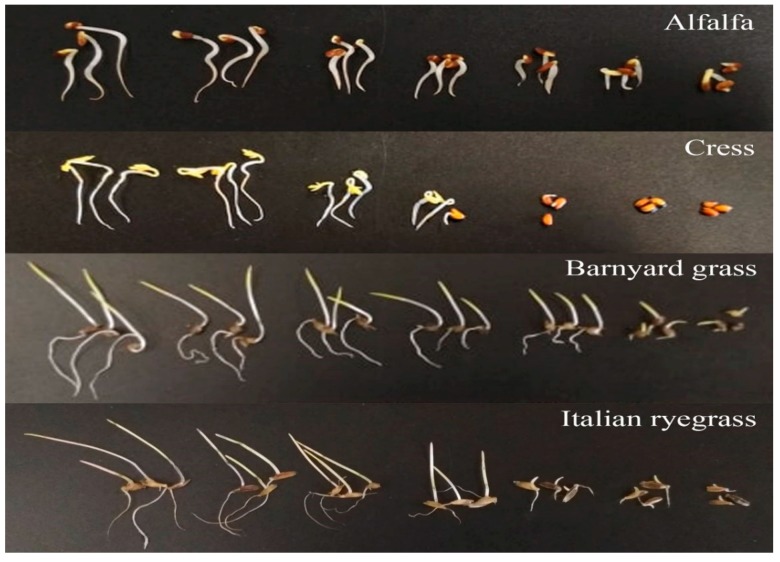
Effect of *S. dichotomus* extracts on the growth of alfalfa, cress, barnyard grass, and Italian ryegrass. Treatment concentrations (from left to right in each picture): control, 0.001, 0.003, 0.01, 0.03, 0.1, and 0.3 g dry weight equivalent extract of *S. dichotomus*/mL.

To evaluate the phytotoxic potential of the leaf extracts of *S. dichotomus*, a growth bioassay was conducted against four test plants: alfalfa, cress, barnyard grass, and Italian ryegrass. The aqueous methanolic extracts of *S. dichotomus* markedly inhibited the shoot and root growth of all the tested plant species. The strength of the phytotoxicity of the extracts greatly varied by concentration and test plant. The growth of all the tested plants declined with increasing extract concentration (Figure 1 and Figure 2). Significant inhibition of all the test plants started with 0.01 g dry weight equivalent extract/mL. At the concentration of 0.1 g dry weight equivalent extract/mL, the shoot and root growth of cress was completely inhibited. When the concentration was increased to 0.3 g dry weight equivalent extract/mL, the shoot lengths of alfalfa, barnyard grass, and Italian rye grass were stunted to 4.5%, 10.0%, and 11.4% of control, respectively. At the same concentration, the root lengths of alfalfa, barnyard grass, and Italian rye grass were reduced to 1.2%, 1.4%, and 2.4% of the control root length, respectively. The *I*_50_ values of the extracts against the test plants ranged from 5.7 to 58.9 mg dry weight equivalent extract/mL (Table 1). Comparing *I*_50_ values, the seedling growth of cress (*I*_50_ = 5.7–7.3 mg dry weight equivalent extract/mL) was most susceptible to inhibition, whereas the shoot growth of barnyard grass (*I*_50_ = 58.9 mg dry weight equivalent extract/mL) and the root growth of Italian rye grass (*I*_50_ = 25.9 mg dry weight equivalent extract/mL) were most resistant to inhibition by the extract of *S. dichotomus*.

### 2.2. Identification of Phytotoxic Compounds and Their Growth-Inhibitory Effect

**Figure 3:**
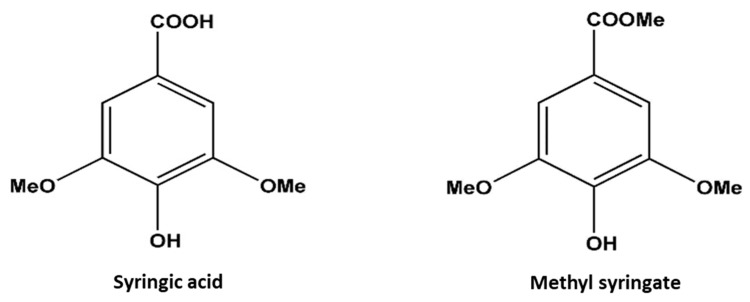
Chemical structures of syringic acid and methyl syringate.

**Figure 4:**
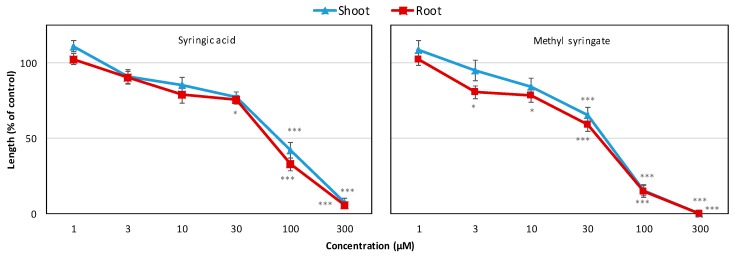
Effects of syringic acid and methyl syringate on the shoot and root growth of cress. Values represent mean ± SE of three replicate Petri dishes for each treatment (n = 30). Significant differences between control and treatment are represented by * *p* < 0.05 and *** *p* < 0.001, respectively.

**Figure 5:**
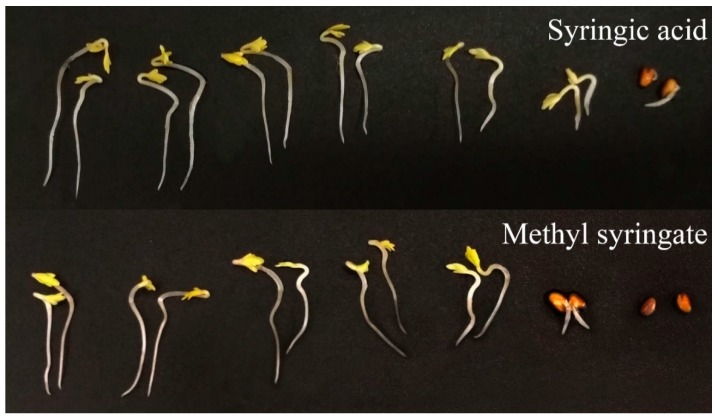
Effects of syringic acid and methyl syringate on the shoot and root growth of cress. Treatment concentrations (from left to right in each picture): control, 1, 3, 10, 30, 100, and 300 µM.

**Figure 6:**
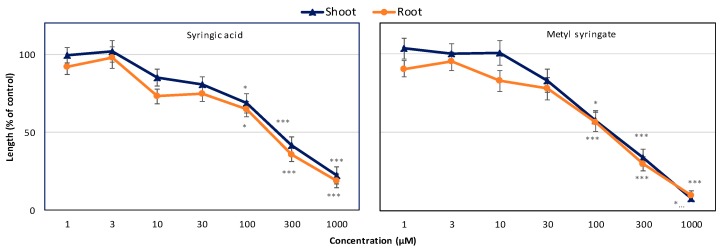
Effects of syringic acid and methyl syringate on the shoot and root growth of Italian ryegrass. Values represent mean ± SE of three replicate Petri dishes for each treatment (n = 30). Significant differences between control and treatment are represented by * *p* < 0.05 and *** *p* < 0.001, respectively.

**Figure 7:**
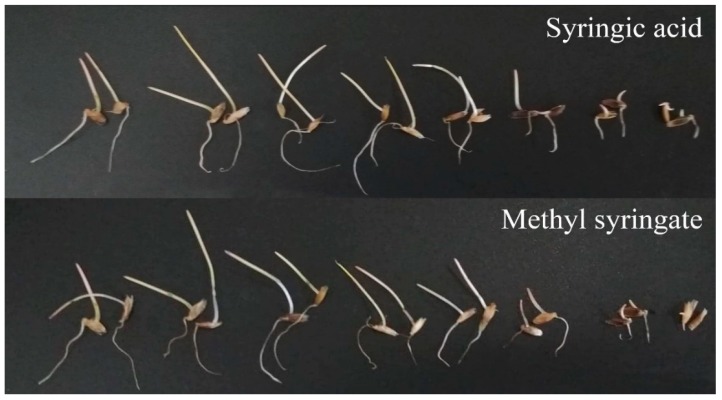
Effects of syringic acid and methyl syringate on the shoot and root growth of Italian rye grass. Treatment concentrations (from left to right in each picture): control, 1, 3, 10, 30, 100, 300, and 1000 µM.

The crude ethyl acetate fraction was repeatedly fractionated over a series of column chromatographic phases using silica gel, Sephadex LH-20, and a C_18_ cartridge, and the fraction was finally purified using reverse-phase HPLC to yield two phytotoxic substances (Compounds **1** and **2**). Compounds **1** and **2** were characterized as syringic acid and methyl syringate, respectively, by comparing the spectral and optical rotation data with previously reported data (Figure 3). Consequently, syringic acid and methyl syringate were assayed against cress and Italian ryegrass to confirm their phytotoxic properties. Data obtained from the assay showed that both compounds severely affected the seedling growth of cress and Italian ryegrass (Figure 4, Figure 5, Figure 6 and Figure 7). The degree of inhibition by the compounds increased with increasing concentration, indicating dose-response behavior. Syringic acid and methyl syringate significantly inhibited the seedling growth of cress at concentrations of 30 and 3 µM, respectively (Figure 4 and Figure 5). At the concentration of 300 µM, methyl syringate completely inhibited the seedling growth of cress, while the same concentration of syringic acid arrested the shoot and root growth of cress to 7.5% and 5.6% of control, respectively. On the other hand, syringic acid and methyl syringate significantly inhibited Italian rye grass at a concentration of 100 µM (Figure 6 and Figure 7). At the highest concentration of 1000 µM, the shoot and root growth of Italian rye grass was inhibited to 22.4%–19.0% and 8.0%–9.8% of the corresponding control treatment, respectively. The *I*_50_ values of syringic acid for the shoot and root growth of cress were 75.8 and 61.3 μM, respectively, and were around 2.8 times higher for Italian ryegrass at 213.6 and 175.8 μM, respectively (Table 2). Likewise, for methyl syringate, the *I*_50_ values were 140.4 and 130.8 μM for Italian rye grass, which were around 3.2 and 4.2 times higher than those values for the cress shoots (43.2 μM) and roots (31.5 μM), respectively. Based on the *I*_50_ values of the compounds, it was evident that methyl syringate had a greater inhibitory effect on both the test plants compared with syringic acid. Moreover, the shoot and root growth of cress showed greater sensitivity than Italian ryegrass to both compounds, and thereby showed species-specific growth-inhibitory activities.

## 3. Discussion

This study showed the phytotoxic effects of aqueous methanol extracts of *S. dichotomus*. The leaf extracts of *S. dichotomus* significantly inhibited the seedling growth of all four tested plant species, alfalfa, cress, barnyard grass, and Italian ryegrass. The phytotoxic activity increased with increasing extract concentration and varied among the target species. Our findings corroborate studies by other researchers [31,32,33], who showed the concentration- and test species-dependent growth-inhibitory effects of different plant extracts. These results indicated that the growth-inhibitory effect might be due to allelopathic substances within the plant extracts.

Purification of the most active fractions following phytotoxicity assays led to isolating and identifying two substances, syringic acid and methyl syringate. Both compounds are synthesized in plants through a series of enzymatic reactions via the shikimic acid pathway [34]. Syringic acid is a naturally occurring phenolic compound present in olives, pumpkin, grapes, and red wine [35,36], and possesses medicinally important properties such as anti-oxidant, anti-microbial, anti-inflammatory, and anti-diabetic activities [37]. On the other hand, methyl syringate is the ester of syringic acid and can be obtained by condensation of the carboxyl group of syringic acid with methanol. Methyl syringate has been previously detected in *Vitis vinifera* vines, *Cestrum parqui* leaves, and *Taraxacum formosanum* roots [38]. It has also been reported as one of the abundant constituents of honey obtained from the nectar of plants such as sunflower, clover, and chestnut [39]. Methyl syringate shows some biological activities, including anti-oxidant and anti-radical activities [40,41]. In addition, it was isolated from *Betula alba* as a strong aflatoxin-inhibitory component [42]. Notably, syringic acid and methyl syringate coexist in manuka honey and are used as chemical markers for a purity test [43]. However, although syringic acid and methyl syringate have been reported in many plants, so far, they have not yet been reported in *S. dichotomus*.

The bioassay results of syringic acid and methyl syringate show that both substances have significant phytotoxic effects against cress and Italian ryegrass. In addition, cress showed higher sensitivity to both compounds compared with Italian ryegrass, and the degree of sensitivity varied by concentration. Several previous studies have also documented such concentration- and testspecies-dependent phytotoxicity of allelochemicals [44,45,46]. The different sensitivities of test species to an allelopathic substance mainly depend on the physiological and biochemical attributes of each test plant. [47]. The growth-inhibitory potentials of syringic acid and methyl syringate are also supported by other researchers, who documented strong phytotoxic activities of these compounds against different standard test plants [48,49,50].

The *I*_50_ values of the substances indicate that methyl syringate showed higher inhibitory activity compared with syringic acid. The difference in phytotoxicity may be due to the dissimilarity of their molecular structures because the phytotoxicity of allelopathic substances depends on their structural differences [51,52,53]. Syringic acid (a derivative of benzoic acid) consists of a single benzene ring with one OH group in the para position, two OCH_3_ groups in the ortho and meta positions, and one COOH group. A study on structure–activity relationships revealed that the number and positions of OH and OCH_3_ groups determine the phytotoxicity of benzoic acids [54]. Moreover, it has been reported that methoxy and hydroxy substituents in the benzene ring increase and decrease the phytotoxicity of benzoic acids, respectively [55]. Recently, some researchers [37] have reported that two methoxy moieties present in the ortho and meta positions of the benzene ring are responsible for the biological activity of syringic acid. Therefore, we assume that the two methoxy groups present in the benzene ring of syringic acid contribute to its phytotoxic potential.

In contrast, the structure of methyl syringate is similar to syringic acid except for an ester group that replaces the COOH group of syringic acid. Methylation of the carboxyl group confers a degree of lipophilicity to methyl syringate compared with the parent acid [56]. In the literature, the phytotoxic effect of phenolic compounds has been related to their lipophilic character. Substances that are more lipophilic tend to be more phytotoxic because they pass more easily through the cell membrane [57]. In addition, it has been proposed that herbicides should be formulated as esters because of their higher solubility in the waxes of the leaf surface, which in turn leads to increased uptake and, hence, phytotoxicity [58]. These studies may explain the higher phytotoxicity of methyl syringate over syringic acid. Moreover, the findings in one study [50] revealed the stronger phytotoxicity of methyl syringate against lettuce germination compared with syringic acid. Therefore, the growth-inhibitory properties of syringic acid and methyl syringate may contribute to the phytotoxic activity of *S. dichotomus*. Thus, it is reasonable to suggest that the phytotoxic properties of *S. dichotomus* enable this plant species to grow successfully with minimum weed infestation because of the suppression of adjacent plants by the release of phytotoxic substances.

## 4. Materials and Methods

### 4.1. Plant Materials

Mature leaves of *Schumannianthus dichotomus* (Roxb.) Gagnep were collected from Sylhet Sadar (24.8917° N, 91.8833° E), Sylhet, Bangladesh in August 2017. The collected leaves were properly washed and shed dried at ambient temperature. The dried leaves were then finely ground into powder using an electric grinder and maintained at 2 °C until use. Two monocot species [barnyard grass (*Echinochloa crus-galli* (L.) P. Beauv.) and Italian ryegrass (*Lolium multiflorum* Lam.)] and two dicot plant species [alfalfa (*Medicago sativa* L.) and cress (*Lepidium sativum* L.)] were chosen as test plants. Cress and alfalfa were selected for their familiar growth patterns, while barnyard grass and Italian ryegrass were selected for their worldwide distribution in crop fields.

### 4.2. Extraction and Bioassay

*S. dichotomus* leaf powder (100 g), obtained from the dried leaves of a single batch, was mixed with 500 mL 70% (*v*/*v*) aqueous methanol as described in previous literature [59]. The mixture was filtered using one layer of filter paper (No. 2, 125 mm; Toyo Ltd., Tokyo, Japan) after two days. An equal volume of cold methanol was added to the residue of the first extraction, kept for one day, and filtered again. Two extracts were then combined and dried in a rotary evaporator at 40 °C. The subsequent crude extract was dissolved in 250 mL methanol to prepare six concentrations (0.001, 0.003, 0.01, 0.03, 0.1, and 0.3 g dry weight equivalent extract/mL) to evaluate the possible phytotoxicity of *S. dichotomus*. These concentrations were obtained by placing an aliquot of methanol extract (1.5, 4.5, 15, 45, 150, and 450 µL) on filter paper (No. 2, 28 mm; Toyo) in 28 mm Petri dishes, which was then dried completely. The filter papers in the Petri dishes were then soaked with 0.6 mL of 0.05% (*v*/*v*) aqueous solution of polyoxyethylene sorbitan monolaurate (Tween 20; Nacalai Tesque, Inc., Kyoto, Japan), and then 10 seeds and 10 seedlings of each of the dicot and monocot test species, respectively, were placed on the filter papers. Simultaneously, control treatments were set up with only 0.6 mL of 0.05% (*v*/*v*) aqueous solution of Tween 20. The dishes were kept in a tray, covered with polyethylene film and aluminum foil, and incubated in a growth chamber (25 °C, darkness). After 48 h of incubation, the lengths of the roots and shoots of those seedlings were measured and compared with the control seedlings.

### 4.3. Purification of the Active Substances

A total of 2 kg powdered *S. dichotomus* sample was extracted as described in the extraction procedure. The extracts were then evaporated using a rotary evaporator (40 °C) to yield a concentrated crude extract. The crude extract was then suspended in distilled water, and the solvent was adjusted to pH 7.0 using 1 M phosphate buffer followed by partitioning four times with an equal volume of ethyl acetate. A growth bioassay was conducted with the obtained aqueous and ethyl acetate fractions against cress as described above. The ethyl acetate fraction showed stronger activity; therefore, a bioassay guided purification procedure [60] was followed for purification of active substances. First, the dried crude mass was separated by chromatography using a silica gel column (60 g of silica gel 60, spherical, 70–230 mesh; Nacalai Tesque, Inc.), and eluted stepwise with *n*-hexane (150 mL per step) containing increasing amounts of ethyl acetate (10% per step, *v*/*v*) from 20% to 80%, ethyl acetate (150 mL per step), and methanol (300 mL per step).

The results from the cress bioassay showed that the fractions obtained with 60% and 70% ethyl acetate in the *n*-hexane possessed the highest biological activity. These two active fractions were then combined and chromatographed in a Sephadex LH-20 column (GE Healthcare Bio-Sciences AB, SE-751 84 Uppsala, Sweden) and stepwise eluted with 20%, 30%, 40%, 50%, 60%, and 80% (*v*/*v*) aqueous methanol (150 mL per step) and methanol (300 mL per step). The highest activity was found with the 40% aqueous methanol fraction, which was then loaded onto a reverse-phase C_18_ cartridge. The cartridge was eluted with 20%, 30%, 40%, 50%, 60%, and 80% (*v*/*v*) aqueous methanol (15 mL per step) and methanol (30 mL per step). The components of the most phytotoxic fraction (obtained with 40% aqueous methanol) were separated using reverse-phase HPLC (500 × 10 mm I.D. ODS AQ-325; YMC Ltd., Kyoto, Japan) at a flow rate of 1.5 mL/min with 45% aqueous methanol (detection wavelength: 220 nm, temperature: 40 °C) to obtain two inhibitory substances. Compounds **1** and **2** were isolated at the retention times of 120–130 and 135–145 min, respectively, and the substances were again purified using reverse-phase HPLC on an ODS column (4.6 × 250 mm I.D., S-5 µm, Inertsil^®^ ODS-3; GL Science Inc., Tokyo, Japan) using 30% aqueous methanol as a mobile phase at a flow rate of 0.8 mL/min. Compounds **1** and **2** were obtained at retention times of 50–60 and 60–70 min, respectively, at a wavelength of 220 nm. The substances were characterized using HRESIMS and ^1^H-NMR.

Finally, comparing obtained spectral data (Table S1) with previously published data [61,62], compounds **1** and **2** were characterized as syringic acid (4-hydroxy-3,5-dimethoxybenzoic acid) and methyl syringate (methyl 4-hydroxy-3,5-dimethoxybenzoate), as shown in Figure 3.

### 4.4. Bioassay of the Isolated Compounds

The isolated compounds were dissolved in 1 mL of methanol and placed in 28 mm Petri dishes containing filter paper to obtain the final assay concentrations of 1, 3, 10, 30, 100, 300, and 1000 μM. After the filter paper was completely dry, it was soaked with 0.6 mL of 0.05% (*v*/*v*) aqueous Tween 20 solution. Ten homogenous seeds of cress were placed in each Petri dish and maintained in a growth chamber under constant darkness at 25 °C. The shoot and root lengths of the cress seedlings were recorded after 48 h of growth and compared with the control seedlings.

### 4.5. Statistics

The bioassay experiment was conducted in a completely randomized design with three replications and two repeats. For all of the assays, SPSS software version 16.0 (SPSS Inc., Chicago, IL, USA) was used for statistical analysis. ANOVA (analysis of variance) followed by Tukey’s test at a significance level of 0.05 was used to evaluate differences among means. The concentration required for 50% growth inhibition (*I*_50_ value) was calculated using a regression equation of the concentration–response curves, and these values were compared for identified compounds by a paired *t*-test.

## 5. Conclusions

The results of this study showed that *S. dichotomus* has strong phytotoxic potential, suppressing the shoot and root growth of alfalfa, cress, barnyard grass, and Italian ryegrass. Two phytotoxic substances, syringic acid and methyl syringate, were isolated from the aqueous methanol extracts of the leaves of *S. dichotomus*. Syringic acid and methyl syringate significantly inhibited the growth of cress and Italian ryegrass. This report is the first on the phytotoxic potential of *S. dichotomus* with suspected phytotoxic substances. Our work indicated the possibility of using *S. dichotomus* as a source to manage weeds.

## Figures and Tables

**Table 1:** The concentration of the extracts of *S. dichotomus* required for 50% growth inhibition (*I*_50_) of the shoot and root growth of each test plant.

Aqueous Methanol Extracts (mg Dry Weight Equivalent Extract/mL)		
Test Plant Species	Shoot	Root
Alfalfa	14.0	11.5
Cress	5.7	7.4
Barnyard grass	58.9	21.1
Italian ryegrass	16.9	25.9

**Table 2:** *I*_50_ values (concentration required for 50% growth inhibition) of the syringic acid and methyl syringate from *S. dichotomus* for shoot and root growth of cress and Italian ryegrass.

Test Plants		Syringic Acid	Methyl Syringate
		(µM)	
Cress	Shoot ***	75.8	43.2
	Root ***	61.3	31.5
Italian ryegrass	Shoot ***	213.6	140.4
	Root ***	175.8	130.8

Significant differences between syringic acid and methyl syringate are represented by *** *p* < 0.001 (paired *t*-test).

## Supplementary Materials

The following are available online at https://www.mdpi.com/2223-7747/9/1/102/s1, Figure S1: Chromatogram of compound **1**, Figure S2: Chromatogram of compound **2**, Table S1: Spectral data of compounds **1** and **2**.

## References

[1] Duke S.O. (1985). Weed Physiology.

[2] Din Z.U., Rodrigues-Filho E., de Cassia Pereira V., Gualtieri S.C.J., Deflon V.M., da Silva Maia P.I., Kuznetsov A.E. (2017). Phytotoxicity, structural and computational analysis of 2-methyl-1,5-diarylpentadienones. J. Mol. Struct..

[3] Seufert V., Mehrabi Z., Gabriel D., Benton T.G., Lemaire G., De Faccio Carvalho P.C., Kronberg S., Recous S. (2019). Current and potential contributions of organic agriculture to diversification of the food production system. Agroecosystem Diversity.

[4] Santos P.C.D., Santos V.H.M.D., Mecina G.F., Andrade A.R.D., Fegueiredo P.A., Moraes V.M.O., Silva R.M.G.D. (2015). Phytotoxicity of *Tagetes erecta* L. and *Tagetes patula* L. on plant germination and growth. S. Afr. J. Bot..

[5] Jabran K., Farooq M., Cheema Z.A., Farooq M., Wahid A. (2013). Implications of potential allelopathic crops in agricultural systems. Allelopathy.

[6] Gonçalves S., Ferraz M., Romano A. (2009). Phytotoxic properties of *Drosophyllum lusitanicum* leaf extracts and its main compound plumbagin. Sci. Hortic..

[7] Jabran K., Mahajan G., Sardana V., Chauhan B.S. (2015). Allelopathy for weed control in agricultural systems. Crop Prot..

[8] Cangiano T., Dellagreca M., Fiorentino A., Isidori M., Monaco P., Zarrelli A. (2002). Effect of ent-labdane diterpenes from Potamogetonaceae on *Selenastrum capricornutum* and other aquatic organisms. J. Chem. Ecol..

[9] Dellagreca M., Isidori M., Lavorgna M., Monaco P., Previtera L., Zarrelli A. (2004). Bioactivity of phenanthrenes from Juncus acutus on *Selenastrum capricornutum*. J. Chem. Ecol..

[10] Pan L., Li X.Z., Yan Z.Q., Guo H.R., Qin B. (2015). Phytotoxicity of umbelliferone and its analogs: Structure–activity relationships and action mechanisms. Plant Physiol. Biochem..

[11] Duke S.O. (2003). Ecophysiological aspects of allelopathy. Planta.

[12] Bogatek R., Gniazdowska A., Zakrzewska W., Oracz K., Gawronski S.W. (2006). Allelopathic effects of sunflower extracts on mustard seed germination and seedling growth. Biol. Plant.

[13] Hachinohe M., Matsumoto H. (2007). Mechanism of selective phytotoxicity of l-3,4-dihydroxyphenylalanine (l-Dopa) in barnyardglass and lettuce. J. Chem. Ecol..

[14] Dastan D., Salehi P., Ghanati F., Gohari A.R., Maroofi H., Alnajar N. (2014). Phytotoxicity and cytotoxicity of disesquiterpene and sesquiterpene coumarins from *Ferula pseudalliacea*. Ind. Crop. Prod..

[15] Duke S.O., Dayan F.E., Romagni J.G., Rimando A.M. (2000). Natural products as sources of herbicides: Current status and future trends. Weed Res..

[16] Céspedes C.L., Salazar J.R., Ariza-Castolo A., Yamaguchi L., Ávila J.G., Aqueveque P., Alarcón J. (2014). Biopesticides from plants: *Calceolaria integrifolia* s.l. Environ. Res..

[17] Demasi S., Caser M., Vanara F., Fogliatto S., Vidotto F., Negre M., Scariot V. (2019). Ailanthone from *Ailanthus altissima* (Mill.) Swingle as potential natural herbicide. Sci. Hortic..

[18] Mandal R.N., Bar R., Chakrabarti P.P. (2014). ‘Pati bet’, *Schumannianthus dichotomus* (Roxb.) Gagnep.—A raw material for preparation of livelihood supporting handicrafts. Indian J. Nat. Prod. Resour..

[19] Rahaman M., Das N.C., Saha N., Islam M. (2010). Nature, profitability and sustainability of murta [*Schumannianthus dichotomus* (Sal.) Willd.] based small-scale enterprises in north-eastern Bangladesh. Small-Scale For..

[20] Hooker J.D. (1874). The Flora of British India.

[21] Joshi S.K., Sharma B.D., Bhatia C.R., Singh R.V., Thakur R.S. (1992). The Wealth of India Raw Materials.

[22] Chowdhury M.S.H., Uddin M.S., Haque F., Muhammed N., Koike M. (2007). Indigenous management of Patipata (*Schumannianthus dichotomus* (Roxb.)) plantation in the rural homesteads of Bangladesh. Subtrop. Agric. Res. Dev..

[23] Mohiuddin M., Rashid M.H. (1988). Survival and growth of vegetatively grown pati-pata (*Schumannianthus dichotomas*): An exploratory study. J. For. Sci..

[24] Prain D. (1903). Bengal Plants.

[25] Rao A.S., Verma D.M. (1972). Materials towards a monocot flora of Asam-II (Zingiberaceae & Marantaceae). Bull. Bot. Sur. India.

[26] Barbhuiya A.H., Ismail K. (2016). Effect of fiber length and loading on the properties of *Schumannianthus dichotomus* (murta) fiber–reinforced epoxy composites. Int. J. Polym. Anal. Charact..

[27] Ahmed R., Islam A.N.M.F., Rahman M., Halim M.A. (2007). Management and economic value of *Schumannianthus dichotoma* in rural homesteads in the Sylhet region of Bangladesh. Int. J. Biodivers. Sci. Manag..

[28] Chowdhury D., Konwar B.K. (2006). Morphophenology and karyotype study of patidoi (*Schumannianthus dichotomus* (Roxb.) Gagnep. synonym *Clinogyne dichotoma* Salisb.)—A traditional plant of Assam. Curr. Sci..

[29] Merry S.R., Ara R., Siddiqi N.A. (1997). Raising seedlings of patipata (*Schumannianthus dichotoma*). J. For. Sci..

[30] Rashid M.H., Merry S.R., Ara R., Mohiuddin Mand Alam M.J. (1993). How to Cultivate Rattan and Patipata.

[31] Appiah K., Mardani H., Omari R., Eziah V., Ofosu-Anim J., Onwona-Agyeman S., Fujii Y. (2018). Involvement of carnosic acid in the phytotoxicity of *Rosmarinus officinalis* leaves. Toxins.

[32] Islam M.S., Iwasaki A., Suenaga K., Kato-Noguchi H. (2017). Isolation and identification of two potential phytotoxic substances from the aquatic fern *Marsilea crenata*. J. Plant Biol..

[33] Rob M.M., Kato-Noguchi H. (2019). Study of the allelopathic activity of *Garcinia pedunculata* Roxb. Plant Omics.

[34] Tohge T., R Fernie A. (2017). An overview of compounds derived from the shikimate and phenylpropanoid pathways and their medicinal importance. Mini Rev. Med. Chem..

[35] Pezzuto J.M. (2008). Grapes and human health: A perspective. J. Agric. Food Chem..

[36] Pacheco-Palencia L.A., Mertens-Talcott S., Talcott S.T. (2008). Chemical composition, antioxidant properties, and thermal stability of a phytochemical enriched oil from acai (*Euterpe oleracea* Mart.). J. Agric. Food Chem..

[37] Srinivasulu C., Ramgopal M., Ramanjaneyulu G., Anuradha C.M., Kumar C.S. (2018). Syringic acid (SA)—A review of its occurrence, biosynthesis, pharmacological and industrial importance. Biomed. Pharmacother..

[38] Tuberoso C.I., Bifulco E., JerkoviĆ I., Caboni P., Cabras P., Floris I. (2009). Methyl syringate: A chemical marker of asphodel (*Asphodelus microcarpus* Salzm. et Viv.) monofloral honey. J. Agric. Food Chem..

[39] Jerković I., Hegić G., Marijanović Z., Bubalo D. (2010). Organic extractives from *Mentha* spp. honey and the bee-stomach: Methyl syringate, vomifoliol, terpenediol I, hotrienol and other compounds. Molecules.

[40] Szwajgier D., Pielecki J., Targoński Z. (2005). Antioxidant activities of cinnamic and benzoic acid derivatives. Acta Sci. Pol. Technol. Aliment..

[41] Sroka Z., Cisowski W. (2003). Hydrogen peroxide scavenging, antioxidant and anti-radical activity of some phenolic acids. Food Chem. Toxicol..

[42] Jermnak U., Yoshinari T., Sugiyama Y., Tsuyuki R., Nagasawa H., Sakuda S. (2012). Isolation of methyl syringate as a specific aflatoxin production inhibitor from the essential oil of *Betula alba* and aflatoxin production inhibitory activities of its related compounds. Int. J. Food Microbiol..

[43] Alvarez-Suarez J.M., Gasparrini M., Forbes-Hernández T.Y., Mazzoni L., Giampieri F. (2014). The composition and biological activity of honey: A focus on Manuka honey. Foods.

[44] Bouknana D., Jodeh S., Sbaa M., Hammouti B., Arabi M., Darmous A., Haboubi K. (2019). A phytotoxic impact of phenolic compounds in olive oil mill wastewater on fenugreek “*Trigonella foenum-graecum*”. Environ. Monit. Assess..

[45] Rob M., Iwasaki A., Suzuki R., Suenaga K., Kato-Noguchi H. (2019). Garcienone, a novel compound involved in allelopathic activity of *Garcinia Xanthochymus* hook. Plants.

[46] Ladhari A., Romanucci V., De Marco A., De Tommaso G., Di Marino C., Di Fabio G., Zarrelli A. (2018). Herbicidal potential of phenolic and cyanogenic glycoside compounds isolated from Mediterranean plants. Ecol. Quest..

[47] Kobayashi K. (2004). Factors affecting phytotoxic activity of allelochemicals in soil. Weed Biol. Manag..

[48] Abbas T., Tanveer A., Khaliq A., Safdar M.E., Nadeem M.A. (2014). Allelopathic effects of aquatic weeds on germination and seedling growth of wheat. Herbologia.

[49] Li Z.H., Wang Q., Ruan X., Pan C.D., Zhang J.C., Jiang D.A., Wang G.G. (2011). Biological activity and quantification of potential autotoxins from *Picea schrenkiana* leaves. Allelopath. J..

[50] D’Abrosca B., DellaGreca M., Fiorentino A., Monaco P., Zarrelli A. (2004). Low molecular weight phenols from the bioactive aqueous fraction of *Cestrum parqui*. J. Agric. Food Chem..

[51] Macías F.A., Marín D., Oliveros-Bastidas A., Molinillo J.M.G. (2006). Optimization of benzoxazinones as natural herbicide models by lipophilicity enhancement. J. Agric. Food Chem..

[52] Maffei M., Bertea C.M., Garneri F., Scannerini S. (1999). Effect of benzoic acid hydroxy-and methoxy-ring substituents during cucumber (*Cucumis sativus* L.) germination. I.: Isocitrate lyase and catalase activity. Plant Sci..

[53] DellaGreca M., Fiorentino A., Monaco P., Previtera L., Temussi F., Zarrelli A. (2003). New dimeric phenanthrenoids from the rhizomes of *Juncus acutus*. Structure determination and antialgal activity. Tetrahedron.

[54] DellaGreca M., Fiorentino A., Monaco P., Previtera L., Zarrelli A. (2002). A new dimeric 9,10-dihydrophenanthrenoid from the rhizome of *Juncus acutus*. Tetrahedron Lett..

[55] Michalowicz J., Duda W. (2007). Phenols sources and toxicity. Pol. J. Environ. Stud..

[56] Weston R.J., Mitchell K.R., Allen K.L. (1999). Antibacterial phenolic components of New Zealand manuka honey. Food Chem..

[57] Pinho I., Lopes D., Martins R., Quina M. (2017). Phytotoxicity assessment of olive mil solid wastes and influence of phenolic compounds. Chemosphere.

[58] Breeze V.G. (1993). Phytotoxicity of Herbicide Vapour. Reviews of Environmental Contamination and Toxicology.

[59] Islam M.S., Kato-Noguchi H. (2016). Allelopathic potential of the weed *Fimbristylis dichotoma* (L.) on four dicotyledonous and four monocotyledonous test plant species. Res. Crop..

[60] Kato-Noguchi H., Suzuki M., Noguchi K., Ohno O., Suenaga K., Laosinwattana C. (2016). A potent phytotoxic substance in *Aglaia odorata* Lour. Chem. Biodivers..

[61] Rosado T., Bernardo P., Koci K., Coelho A.V., Robalo M.P., Martins L.O. (2012). Methyl syringate: An efficient phenolic mediator for bacterial and fungal laccases. Bioresour. Technol..

[62] Liao C.R., Kuo Y.H., Ho Y.L., Wang C.Y., Yang C.S., Lin C.W., Chang Y.S. (2014). Studies on cytotoxic constituents from the leaves of *Elaeagnus oldhamii* Maxim. in non-small cell lung cancer a549 cells. Molecules.

