# Supplementary material for: Phytotoxic Activity and Identification of Phytotoxic Substances from Schumannianthus dichotomus
Source: Plants (Basel). 2020 Jan 14;9(1):102. doi: 10.3390/plants9010102 (PMC7020185; doi:10.3390/plants9010102)
Supplement: Supplementary file 1 [file plants-09-00102-s001.pdf]

plants

MDPI

Article

Phytotoxic Activity and Identification of Phytotoxic Substances from *Schumannianthus dichotomus*

Md. Mahfuzur Rob [1,3,*], Kawsar Hossen [1,3], Arihiro Iwasaki [2], Kiyotake Suenaga [2] and Hisashi Kato-Noguchi [1]

[1] Department of Applied Biological Science, Faculty of Agriculture, Kagawa University, Miki, Kagawa 761-0795, Japan; mahfuzrob@gmail.com (M.M.R.); kwsarbau@gmail.com (K.H.); hisashi@ag.kagawa-u.ac.jp (H.K.N)

[2] Department of Chemistry, Faculty of Science and Technology, Keio University, 3-14-1 Hiyoshi, Kohoku, Yokohama 223-8522, Japan; a.iwasaki@chem.keio.ac.jp (A.I); suenaga@chem.keio.ac.jp (K.S)

[3] The United Graduate School of Agricultural Sciences, Ehime University, 3-5-7 Tarumi, Matsuyama, Ehime 790-8566, Japan

*Correspondence: mahfuzrob@gmail.com; Tel.: 090-8286-2561, Japan (M.M.R)

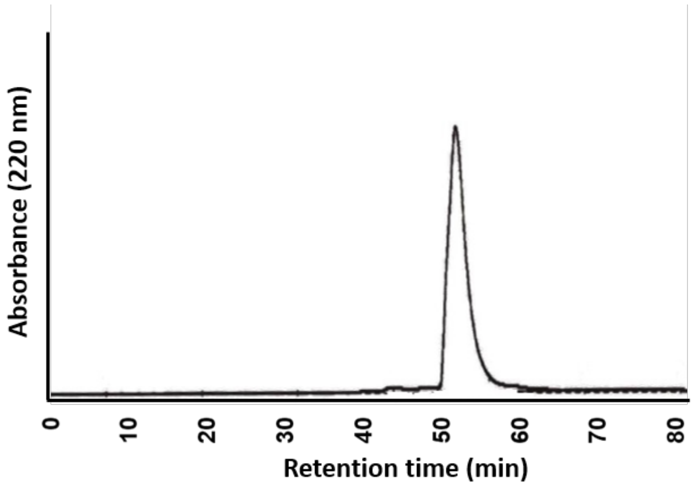

Figure S1. Chromatogram of compound **1** obtained at 50–60 minutes retention time purified by reverse phase HPLC, eluted at a flow rate of 0.8 mL/min with 30% aqueous methanol, detected at 220 nm wavelength.

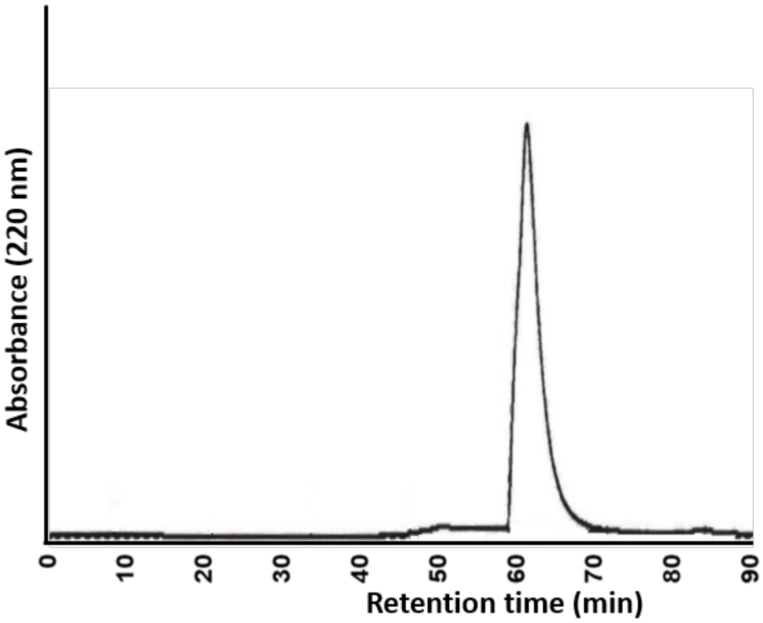

Figure S2. Chromatogram of compound **2** obtained at 60–70 minutes retention time purified by reverse phase HPLC, eluted at a flow rate of 0.8 mL/min with 30% aqueous methanol, detected at 220 nm wavelength.

Table S1. Spectral data of compounds **1** and **2**

Compound Name	Spectral Data
Compound **1**	HRESIMS: *m/z* 199.0300 $[M+H]^+$ (calculated for $C_{12}H_{13}O_5$, 199.0607, $\Delta = -30.7$ mmu); ^{1}H NMR (400 MHz, CD_3OD) δ_H 7.32 (s, 2H, H2,6), 3.91 (s, 6H, H8,9).
Compound **2**	HRESIMS: *m/z* 213.0750 $[M+H]^+$ (calculated for $C_{12}H_{13}O_5$, 213.0763, $\Delta = -1.3$ mmu); ^{1}H NMR (400 MHz, CD_3OD) δ_H 7.32 (s, 2H, H2,6), 3.89 (s, 6H, H8,9), 3.87 (s, 3 H, H10).
